# Comment on “Sitagliptin, a DPP‐4 Inhibitor, Effectively Promotes the Healing of Diabetic Foot Ulcer: A Randomized Controlled Trial”

**DOI:** 10.1111/1753-0407.70165

**Published:** 2025-11-05

**Authors:** Sabika Ayesha, Umaima Parveen, Noor Un Nisa Irshad

**Affiliations:** ^1^ Department of Medicine Jinnah Sindh Medical University Karachi Pakistan

Dear Editor,

1

The study conducted by Gao et al. offers valuable evidence regarding the therapeutic potential of sitagliptin in the management of diabetic foot ulcers (DFUs) [1]. The authors have identified opportunities for adjunctive therapies in a condition that continues to be a morbidity by demonstrating enhancements in ulcer healing and endothelial progenitor cell (EPC) mobilization. Nonetheless, several methodological and interpretative considerations need discussion to place these results in a broader clinical context.

A major concern is the trial's inadequate blinding. The authors state that “blinding was applied exclusively to the personnel responsible for performing area calculations and laboratory evaluator evaluations,” yet patients and treating clinicians were aware of allocation because no placebo was used. This is not a true double‐blind study and risks performance and observer bias. In double‐blind randomized controlled trials, such as the protocol by O'Reilly et al. [2], both patients and clinicians are blinded to ensure wound care and unbiased outcome assessment. Awareness of sitagliptin could have influenced wound care intensity, follow‐up decisions, or judgments of healing, exaggerating the benefit.

Second, the lack of standardized conventional therapy further complicates interpretation. “Conventional therapy” was described broadly to include vasodilators, neurotrophic agents, antibiotics, and wound debridement, but administration was left to the clinician's discretion. In contrast to O'Reilly et al., where standard wound care methods were routinely implemented, such diversity presents significant confounding factors. If the sitagliptin group received more consistent background therapy, observed differences in healing may reflect uneven supportive care rather than a true pharmacologic effect.

Third, the outcome definition is limited. Ulcer healing was defined strictly as 100% area reduction, with substantial partial improvements (e.g., 80%–99%) excluded from the primary endpoint. This all‐or‐nothing approach neglects clinically meaningful outcomes. As Gottrup et al. emphasized [3], wound‐healing studies should also consider infection, pain, resource use, and cost‐effectiveness. Moreover, while Gao et al. reported CD34+ and SDF‐1α changes, reliance on surrogate endpoints is insufficient. Pichu et al. review that biomarker data alone cannot substitute for patient‐centered outcomes or hard endpoints such as amputation or recurrence [4].

Finally, the short follow‐up duration represents a significant methodological flaw. Follow‐up was capped at 12 weeks or until complete healing. However, it frequently takes several months for Wagner Grades 3–4 ulcers to close. In order to evaluate healing, safety, and amputation outcomes, patients with advanced DFUs treated with cryopreserved umbilical cord were monitored for a year in the Marston et al. study [5]. Limiting observation to 12 has the risk of censoring bias, where ulcers that do not heal within this short time frame are incorrectly labeled as “ineffective.” This truncation may restrict long‐term reliability and understate actual healing potential.

To sum up, Gao et al. provide important evidence that hastens DFU healing. Blinding, background therapy standardization, outcome measures, and follow‐up duration limitations, however, may limit generalizability and overstate efficacy. To determine sitagliptin's actual function in the treatment of DFU, more thorough randomized controlled trials with strict blinding, standardized care, and extended follow‐up are required.

## Author Contributions

Conceptualization: Sabika Ayesha, Umaima Parveen. Data curation: Sabika Ayesha, Umaima Parveen. Writing original draft preparation: Sabika Ayesha, Umaima Parveen. Investigation, supervision, and writing review: Noor Un Nisa Irshad. All authors read and approve the final manuscript.

## Ethics Statement

The authors have nothing to report.

## Consent

The authors have nothing to report.

## Conflicts of Interest

The authors declare no conflicts of interest.

## Data Availability

Data sharing not applicable to this article as no datasets were generated or analyzed during the current study.
